# Role of the halo sign in the assessment of giant cell arteritis: a systematic review and meta-analysis

**DOI:** 10.1093/rap/rkab059

**Published:** 2021-08-19

**Authors:** Alwin Sebastian, Fiona Coath, Sue Innes, Jo Jackson, Kornelis S M van der Geest, Bhaskar Dasgupta

**Affiliations:** 1 Department of Rheumatology, Southend University Hospital, Mid and South Essex University Hospital Groups, Westcliff-On-Sea; 2 School of Sport, Rehabilitation and Exercise Sciences, University of Essex, Colchester, Essex; 3 Department of Rheumatology, Norfolk and Norwich University Hospitals NHS Foundation Trust, Norwich, Norfolk, UK; 4 Department of Rheumatology and Clinical Immunology, University Medical Centre Groningen, University of Groningen, Groningen, The Netherlands

**Keywords:** giant cell arteritis, ultrasound, halo sign, glucocorticoids, systematic review

## Abstract

**Objectives:**

This systematic review and meta-analysis aimed to evaluate the diagnostic value of the halo sign in the assessment of GCA.

**Methods:**

A systematic literature review was performed using MEDLINE, EMBASE and Cochrane central register databases up to August 2020. Studies informing on the sensitivity and specificity of the US halo sign for GCA (index test) were selected. Studies with a minimum of five participants were included. Study articles using clinical criteria, imaging such as PET-CT and/or temporal artery biopsy (TAB) as the reference standards were selected. Meta-analysis was conducted with a bivariate model.

**Results:**

The initial search yielded 4023 studies. Twenty-three studies (patients *n* = 2711) met the inclusion criteria. Prospective (11 studies) and retrospective (12 studies) studies in academic and non-academic centres were included. Using clinical diagnosis as the standard (18 studies) yielded a pooled sensitivity of 67% (95% CI: 51, 80) and a specificity of 95% (95% CI: 89, 98%). This gave a positive and negative likelihood ratio for the diagnosis of GCA of 14.2 (95% CI: 5.7, 35.5) and 0.375 (95% CI: 0.22, 0.54), respectively. Using TAB as the standard (15 studies) yielded a pooled sensitivity of 63% (95% CI: 50, 75) and a specificity of 90% (95% CI: 81, 95).

**Conclusion:**

The US halo sign is a sensitive and specific approach for GCA assessment and plays a pivotal role in diagnosis of GCA in routine clinical practice.

**Registration:**

PROSPERO 2020 CRD42020202179.

Key messages

- Compared with previous meta-analyses, the halo sign had similar sensitivity (67%) but higher specificity (95%).
- Higher specificity might potentially reflect improved technique and equipment.
- Studies showed design heterogenicity; we recommend that future researchers adopt multicentre prospective standardized study protocols.

## Introduction

GCA is a form of large vessel vasculitis, which can cause critical ischaemia. Associated retinal ischaemia can lead to permanent blindness in ∼15–25% of patients, making it a medical emergency [1]. However, making a diagnosis of GCA can be challenging, because none of the symptoms or laboratory findings have perfect sensitivity or specificity for the disease [2]. The ACR 1990 classification criteria for GCA have been developed for research purposes, but have limited specificity for GCA in daily clinical practice [3].

Since the publication of the ACR Classification criteria, ultrasonography has been shown to play a pivotal role in the diagnosis of GCA, with the most specific finding being the halo sign, a circumferential hypoechoic vessel wall thickening around the lumen, most probably attributable to vessel wall oedema [4] and intimal hyperplasia [5]. GCA predominantly involves the external carotid artery and its branches, such as the temporal arteries (cranial GCA), the aorta, subclavian and axillary arteries [6]. Traditionally, glucocorticoids (GCs) have been the mainstay of treatment for GCA [7], although cohort studies and the GiACTA trial showed only 15–20% sustained remission with GCs alone [8]. Current guidelines suggest starting GCs immediately in patients where GCA is strongly suspected, pending investigation, to prevent serious ischaemic complications [9]. Long-term use of high-dose GCs can lead to severe adverse effects, such as hypertension, hyperglycaemia, osteoporosis, Cushingoid changes, mood disturbance, electrolyte imbalance, cataracts and glaucoma, but this is not an exhaustive list [9, 10]. Therefore, a prompt and accurate diagnosis is vital to ensure that vision is preserved whilst avoiding unnecessary exposure to a potentially toxic treatment [11]. GCA fast-track clinics have been shown to reduce permanent visual loss by facilitating a rapid specialist clinical assessment with US of the temporal and/or axillary arteries [1, 12].

Historically, a positive temporal artery biopsy (TAB) has been the gold standard test for a histological diagnosis of GCA [13, 14]. However, TAB is invasive and lacks sensitivity [15]. This deficiency is particularly true with extra-cranial involvement, where access to histological samples has obvious practical constraints and is usually identified incidentally following cardiovascular surgery [15]. Non-invasive imaging techniques, including US, MRI and PET-CT, are readily able to identify these patients [2, 16, 17]. The EULAR recommends US of temporal and/or axillary arteries as the first imaging modality for suspected predominantly cranial GCA, where adequate expertise and equipment are available [18]. US is safe, non-invasive and has high sensitivity. It is a relatively quick procedure, often used as a point-of-care test, well tolerated by patients, with a growing body of evidence for its use in follow-up [19]. At present, a non-compressible halo sign is the main finding on US of active GCA patients [4, 13, 20]. The accuracy and criterion validity of US in the diagnosis of GCA was investigated in several studies [19, 21–23]. A meta-analysis of prospective studies compared the final diagnosis of GCA with temporal artery US, showing a pooled sensitivity of 77% and a pooled specificity of 96% [24].

US also allows for assessment of the intimal media complex and measurement of intimal medial thickness (IMT). Although no definite consensus has been reached, studies suggest that at the age of 70 years, a normal temporal artery has an IMT of ∼0.2 mm, whereas abnormal or inflamed temporal arteries have an IMT range between 0.5 and 0.9 mm [18, 25]. Axillary arteries of patients aged ∼70 years have a normal IMT of ∼0.6 mm, whereas patients with extra-cranial (large vessel) GCA have a mean IMT of 1.6–1.7 mm [25, 26]. An axillary artery IMT of 1.0 mm was determined as a cut-off value to discriminate between a normal and abnormal artery by Schäfer *et al.* [25]. Currently, US assessment of suspected GCA patients is reported in a dichotomous manner (positive or negative). However, a range of extent and severity of these findings can be observed in the temporal and axillary arteries [27]. A recent *post hoc* prospective study of a quantitative ultrasonographic halo score, which combines the grade and extent of halos seen in temporal arteries, their branches and axillary arteries in GCA, has shown value as a marker of disease activity and ocular ischaemia [3]. Whether the halo score might be of help with diagnosis, prognosis and GCA monitoring is being tested in an ongoing prospective multicentre study of patients presenting with new GCA (HAS-GCA study; NIHR IRAS# 264294) [13].

This systematic review and meta-analysis focused on evaluation of the clinical role of the halo sign in managing a clinically suspected GCA population and ascertaining the areas that warrant further exploration. This study also updates estimates of diagnostic accuracy, because newer studies have been published using modern US equipment.

## Methods

For this literature review and meta-analysis, we followed the format of population, intervention, comparator and outcome (PICO) [28] (Supplementary Table S1, available at *Rheumatology Advances in Practice* online) and guidelines of (PRISMA-DTA) Preferred Reporting Items for Systematic Reviews and Meta-Analyses [29, 30]. This study protocol was registered with the international prospective register of systematic reviews (PROSPERO 2020 CRD42020202179). No ethical approval or informed consent was required.

### Literature search

The literature was searched systematically by two investigators (A.S. and F.C.) using a broad search of different databases; MEDLINE, EMBASE and Cochrane central registry (Supplementary Table S2, available at *Rheumatology Advances in Practice* online). These databases were searched for original primary studies that examined the sensitivity and specificity of the halo sign, demonstrated by temporal artery and/or axillary artery ultrasonography for GCA diagnosis, published in English, from their inception dates until August 2020. The search terms included giant cell arteritis, temporal arteritis, diagnostic imaging, imaging, ultrasound, ultrasonography, halo sign and temporal artery biopsy. An experienced medical librarian carried out the complete search.

### Study selection and eligibility criteria

The titles and abstracts were screened by two independent reviewers (A.S. and F.C.). Full texts were assessed independently by two reviewers (A.S. and F.C.). Any disagreement between reviewers was resolved by consensus or, if consensus could not be obtained, by consulting a third reviewer (K.S.M.v.d.G.), who made the final decision.

We included prospective and retrospective cross-sectional or longitudinal studies and randomized controlled trials of GCA, conducted in single or multicentre settings, provided the patients had temporal and/or axillary artery US performed for diagnosis. We included studies: (1) containing patients with suspected GCA; (2) using clinical diagnosis, an imaging test (US/PET-CT) and/or TAB as the reference standard for GCA; (3) in which US was performed at any time from the clinical suspicion of GCA; and (4) in which at least five patients had GCA and at least five did not have GCA. Case reports, case series, conference abstracts and case–control studies were excluded because specificity could not be evaluated. Adult human subjects (age ≥50 years), clinically classified as suspected GCA, were included. The reference standard clinical diagnosis of GCA was considered when the treating clinician-suspected GCA based on clinical criteria such as age ≥50 years, abnormal blood markers (CRP >5 mg/l, ESR >30 mm/h), unequivocal cranial symptoms of GCA and/or PMR symptoms and evidence of GCA by imaging (US/PET-CT) or positive TAB. All the participants must have had a temporal artery and/or axillary artery US to look for the halo sign and/or compression sign, occlusion and stenosis. Moreover, TAB was also used as a reference standard separately.

### Data collection

Study characteristics and data from 2 × 2 tables (true positive, false negative, false positive or true negative) were extracted by one reviewer (A.S.) and checked by a second reviewer (F.C.). If no consensus could be obtained, a third reviewer (K.S.M.v.d.G.) made the final decision. A standard data sheet was used to collect information on study characteristics. Authors of studies were not contacted. In the event of potential overlap of patients between studies from the same hospital, data were obtained from the most extensive study for the meta-analysis. When multiple reference standards were used in the same study, the clinical diagnosis was used as the primary reference standard for the data analysis. The other was used for sub-group analysis. Any disagreement between reviewers was either resolved by consensus or by consulting a third reviewer (K.S.M.v.d.G.).

### Quality assessment

The risk of bias was evaluated by two reviewers (A.S. and F.C.) with the quality assessment of diagnostic accuracy studies (QUADAS-2) tool [31]. Any disagreement between reviewers was resolved through discussion with other review authors (S.I., J.J. and B.D.). The QUADAS-2 tool focuses on the bias and applicability of study results regarding patient selection, the index test, the reference standard, and study flow and timing [31].

### Statistical analysis

The sensitivity and specificity of the halo sign, along with their 95% CIs, were calculated for each study, and the total sample size of reviews was plotted. Study heterogeneity was examined visually by plotting sensitivity and specificity in forest plots and receiver operating characteristics (ROC) space [32]. We used hierarchical logistic regression modelling (bivariate model) (Supplementary Fig. S1, available at *Rheumatology Advances in Practice* online) to determine pooled estimates of diagnostic accuracy parameters (i.e. sensitivity, specificity, diagnostic odds ratio and likelihood ratios). Stata v.15 software was used for the statistical analysis and creating hierarchical summary receiver-operating characteristic (HSROC) plots. Forest plots were created in Review Manager v.5.3.

## Results

### Study characteristics

**Fig. 1:**
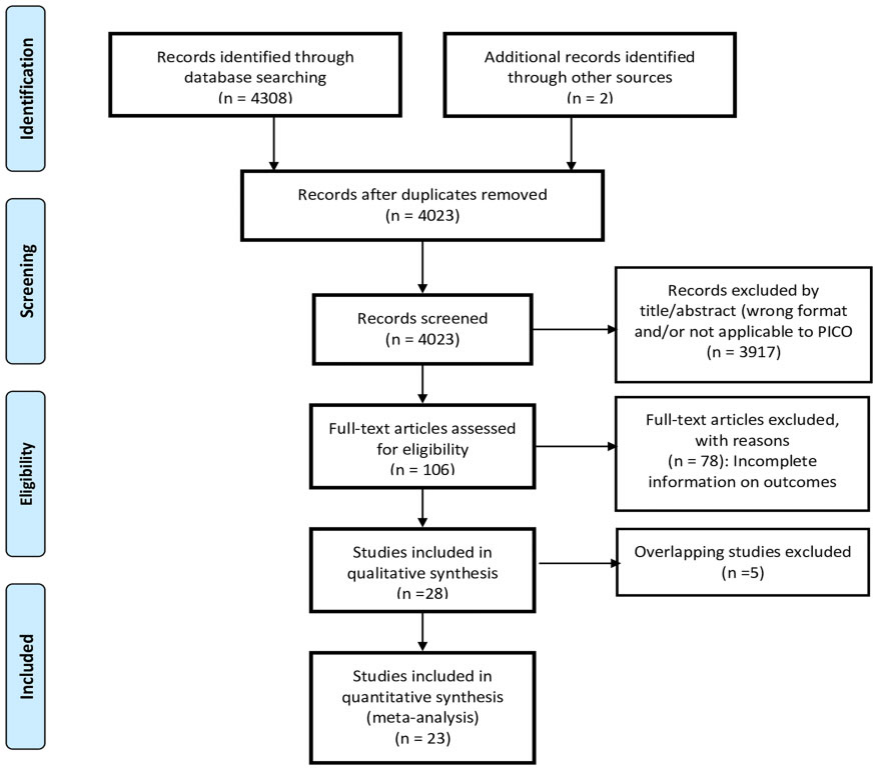
PRISMA flow diagram

The initial search yielded 4023 unique studies. Based on title/abstract screening, 106 articles were selected for full-text screening. Twenty-three articles were selected for the systematic review and meta-analysis [15, 24, 33–53]. The flow of information through the review is illustrated in the PRISMA flow diagram [54, 55] (Fig. 1).

A total of 2711 subjects were collected from 23 studies, and their characteristics are summarized in the study characteristics table (Table 1). There were 12 retrospective and 11 prospective studies performed at academic and non-academic centres. Clinical diagnosis was the most commonly used reference standard, whereas some reports presented TAB as the reference standard. A variable proportion of patients underwent unilateral or bilateral temporal artery US assessment (Table 2). The clinical diagnosis was based mainly on clinical and laboratory findings, imaging and/or TAB results. In the studies using clinical diagnosis as a reference standard (18 studies), all patients were reviewed to ensure the clinical diagnosis was not later revised. The majority of studies assessed the cranial arteries alone (15 studies), whereas others evaluated both cranial and extra-cranial arteries (eight studies). Most of the GCA studies tested the halo sign as a main lesion to define vasculitis. Other US signs addressed (mostly in combination with the halo sign) were stenosis and occlusion [33, 39, 43, 50] and the compression sign [24, 40]. Two studies reported the compression sign [24, 40], and four studies reported stenosis and occlusion along with halo sign [33, 39, 43, 50]. Fifteen studies used TAB [15, 33, 34, 39, 41–46, 48–50, 52, 53], and two studies used the compression sign [24, 40] as reference. More than half of the publications examined colour duplex US with frequencies of 5–15 MHz. The US specifications are summarized in Table 2.

**Table 1:** Study characteristics and general information

Author (year) (number of patients)	Journal	Period of patients' inclusion	Study design	Hospital setting	Speciality identifying patients	Speciality referring patients	Included patients (patients undergoing)	Conse cutive patients	Avoiding case- control	Lab results reported before treatment	Type of reference standard	Test performed in every patient with clinical diagnosis	**Focus diagnostic testing** **(arteries)**
Schmidt *et al.* (1997) [43] (*n* = 112)	*Rheumatology*	January 1994 to October 1996	Retrospective	Academic	Ophthalmology department	Primary care and hospital departments	TAB	Yes	Yes	Unclear	Clinical diagnosis (+TAB)	TAB, US	Cranial
Nesher *et al.* (2002) [52] (*n* = 69)	*The Journal of Rheumatology*	Unclear (2-year time span)	Prospective	Academic	Central imaging registry	Unclear	US	Yes	Yes	Yes	Clinical diagnosis (+TAB)	TAB, US	Cranial
Salvarani *et al.* (2002) [48] (*n* = 86)	*Annals of Internal Medicine*	January 1998 to October 1999	Prospective	Academic	Central pathology/surgery registry	Unclear	TAB	Yes	Yes	Unclear	Clinical diagnosis (+TAB)	TAB, PET-CT, CTA	Cranial and extra-cranial
Lesar *et al.* (2002) [46] (*n* = 32)	*Journal of Vascular surgery*	November 1997 to April 2001	Prospective	Academic	Central imaging registry	Unclear	US	Yes	Yes	NA	TAB	TAB, US	Cranial
Reinhard *et al.* (2004) [49] (*n* = 83)	*Clinical and Experimental Rheumatology*	July 1999 to July 2002	Prospective	Academic	Multiple hospital departments	Unclear	Clinical evaluation	Yes	Yes	NA	Clinical diagnosis (+TAB)	TAB, US	Cranial
Romera-Villegas *et al.* (2004) [42] (*n* = 68)	*Clinical Rheumatology*	May 1998 to November 2002	Retrospective	Academic	Central pathology/surgery registry	Unclear	TAB	Yes	Yes	Unclear	TAB	TAB, US	Cranial
Karahaliou *et al.* (2006) [35] (*n* = 55)	*Arthritis Research & Therapy*	2000–2004	Prospective	Academic	Multiple hospital departments	Unclear	Clinical evaluation	Yes	Yes	Unclear	Clinical diagnosis	US	Cranial
Lopez *et al.* (2009) [34] (*n* = 47)	*Clinical and Experimental Rheumatology*	March 2003 to July 2006	Retrospective	Academic	Central pathology/surgery registry	Unclear	TAB	Yes	Yes	NA	Clinical diagnosis (+TAB)	TAB	Cranial
Maldini *et al.* (2010) [33] (*n* = 31)	*Journal of Nuclear Medicine*	January 2002 to September 2008	Retrospective	Academic	Central imaging registry	Unclear	PET	Yes	Yes	Unclear	Clinical diagnosis (+TAB)	TAB	Cranial and extra-cranial
Pfenninger *et al.* (2012) [44] (*n* = 57)	*Journal of Rheumatology*	January 1999 to February 2011	Retrospective	Non-academic	Central pathology/surgery registry	Unclear	TAB	Yes	Yes	Unclear	TAB	TAB	Cranial
Aschwanden *et al.* (2012) [24] (*n* = 80)	*Ultraschall in der Medizin*	March 2009 to September 2011	Prospective	Academic	Multiple hospital departments	Unclear	US	Yes	Yes	NA	Clinical diagnosis	US	Cranial
Black *et al.* (2013) [38] (*n* = 50)	*International Journal of Rheumatic diseases*	September 2003 to September 2011	Retrospective	Academic	Central imaging registry	Primary care and hospital department	US	Yes	Yes	NA	Clinical diagnosis	US	Cranial
Muratore *et al.* (2013) [45] (*n* = 160)	*British Journal of Rheumatology*	2002–2010	Retrospective	Academic	Central pathology/surgery registry	Primary care	TAB	Yes	Yes	Unclear	TAB	TAB	Cranial
Aschwanden *et al.* (2015) [40] (*n* = 60)	*Clinical and Experimental Rheumatology*	October 2011 to December 2012	Prospective	Academic	Multiple hospital departments	Unclear	clinical; evaluation	Yes	Yes	NA	Clinical diagnosis	US	Cranial
Croft *et al.* (2015) [37] (*n* = 87)	*Journal of the Royal College of Physicians of Edinburgh*	January 2005 to January 2014	Retrospective	Academic	Central imaging registry	Unclear	US	Yes	Yes	NA	Clinical diagnosis	US	Cranial and extra-cranial
Luqmani *et al.* (2016) [15] (*n* = 381)	*Health Technology Assessment*	June 2010 to July 2016	Prospective	Non-academic and academic	Multiple hospital departments	Unclear	TAB	Yes	Yes	Yes	Clinical diagnosis (+TAB)	TAB, US	Cranial and extra-cranial
Bilyk *et al.* (2017) [39] (*n* = 71)	*American Ophthalmological society*	2017 (14 months)	Retrospective	Academic	Central imaging registry	Unclear	US	YES	Yes	NA	Clinical diagnosis (+TAB)	TAB, US	Cranial and extra-cranial
Porto *et al.* (2018) [50] (*n* = 56)	*Rheumatology clinica*	February 2015 to July 2016	Prospective	Academic	Central pathology/surgery registry	Unclear	TAB	Yes	Yes	Unclear	Clinical diagnosis (+TAB)	TAB, US	Cranial
Nielsen *et al.* (2019) [51] (*n* = 90)	*Rheumatology*	October 2014 to June 2018	Prospective	Academic	Rheumatology department	Unclear	Clinical evaluation	Yes	Yes	NA	Clinical diagnosis	PET-CT, US	Cranial and extra-cranial
Sammel *et al.* (2019) [47] (*n* = 6)	*Rheumatology*	May 2016 to July 2018	Prospective	Academic	Rheumatology department	Unclear	Clinical evaluation	Yes	Yes	NA	Clinical diagnosis	US	Cranial
Sommer *et al.* (2019) [41] (*n* = 68)	*Clinical and Experimental Ophthalmology*	2015–2017	Retrospective	Academic	Ophthalmology department	Hospital department	TAB	Yes	Yes	Unclear	TAB	TAB	Cranial
Mukhtyar *et al.* (2019) [53] (*n* = 25)	*Clinical Rheumatology*	March 2013	Retrospective	Academic	Multiple hospital departments	Unclear	TAB and US	Yes	Yes	Yes	Clinical diagnosis (+TAB)	TAB, US	Cranial and extra-cranial arteries
Hop *et al.* (2020) [36] (*n* = 113)	*Rheumatology*	January 2013 to November 2017	Retrospective	Academic	Central imaging registry	Unclear	US	Yes	Yes	Unclear	Clinical diagnosis	US	Cranial and extra-cranial arteries

TAB: temporal artery biopsy.

**Table 2:** US specifications

Author (year)	Manufacturer	Equipment model	Type of probe	Probe frequency (MHz)	Unilateral/bilateral TA assessment	Axillary artery assessment	Index test	Halo thickness	Time from clinical assessment to US
Schmidt *et al.* (1997) [43]	ATL Bothell	Ultramark 9HDI	Linear	5–10	Bilateral	Yes	Halo, stenosis/occlusion	Yes	10 days
Nesher *et al.* (2002) [52]	Acuson	Sequoia 512	Linear	15–8	Uni/bilateral	No	Halo	Yes	3 days
Salvarani *et al.* (2002) [48]	Àcuson	Aspen	Linear	5–10	Bilateral	No	Halo	Yes	NA
Lesar *et al.* (2002) [46]	ATL Ultrasound	ATL 5000	Linear	7–5	Uni/bilateral	No	Halo, stenosis	NA	NA
Reinhard *et al.* (2004) [49]	ATL, Bothell	HDI 5000	Linear	5–10	Unilateral	No	Halo	NA	6 days
Romera-Villegas *et al.* (2004) [42]	Philip Bothell	HDI 5000	Linear	5–10	Unilateral	Yes	Halo	NA	NA
Karahaliou *et al.* (2006) [35]	General Electric	LA39	Linear array	7–10	Uni/bilateral	Yes	Halo, stenosis	Yes	3 months
Lopez *et al.* (2009) [34]	Toshiba	Aplio-80	Linear	5–10	Uni/bilateral	No	Halo, stenosis	Yes	1–10 days
Maldini *et al.* (2010) [33]	ATLToshiba	Apogee 800/Aplio 80	Pencil probe	5/7.5	Uni/bilateral	No	Halo, stenosis/occlusion	BA	30 days
Pfenninger *et al.* (2012) [44]	Toshiba	Aplio 80 (SSA-770)	Linear	5–10	Uni/bilateral	Yes	Halo	Yes	6 months
Aschwanden *et al.* (2012) [24]	Philips, Best, The Netherlands	iU22 Duplex	Linear	5–17	Uni/bilateral	No	Halo/compression	NA	NA
Black *et al.* (2013) [38]	Philips HDI, 5000 Philips IU22	iU22 Duplex	Linear	17	Uni/bilateral	No	Halo	NA	NA
Muratore *et al.* (2013) [45]	ATL Ultrasound	ATL HDI 5000	Linear	7–5	Uni/bilateral	No	Halo, stenosis	Yes	NA
Aschwanden *et al.* (2015) [40]	Philips Best, Netherlands	Iu22 Duplex	Linear	5–17	Uni/bilateral	No	Halo/compression	Yes	NA
Croft *et al.* (2015) [37]	Hitachi Medical systems	Hitachi HA700	Multi-D linear	13–5	Uni/bilateral	No	Halo	Yes	3 months
Luqmani *et al.* (2016) [15]	NA	NA	Multi-D linear	10/6	Uni/bilateral	Yes	Halo	Yes	10 days
Bilyk *et al.* (2017) [39]	Mylab Twice	LA435	Multi-D linear	22–12.5	Uni/bilateral	Yes	Halo, stenosis/occlusion	Yes	NA
Porto *et al.* (2018) [50]	Mindray Z6	Mindray Z6	A7L4P linear	5–10	Uni/bilateral	No	Halo, stenosis/occlusion	NA	3 months
Nielsen *et al.* (2019) [51]	Hitachi	HI VISION Avius	EUP-L75	5–18	Uni/bilateral	Yes	Halo	Yes	3 months
Sammel *et al.* (2019) [47]	NA	NA	NA	NA	Uni/bilateral	No	NA	NA	NA
Sommer *et al.* (2019) [41]	Philips	Affiniti	Linear	5–10	Bilateral	No	NA	NA	NA
Mukhtyar *et al.* (2019) [53]	Toshiba	Viamo	Linear	4–14	Uni/bilateral	No	Halo	Yes	7 days
Hop *et al.* (2020) [36]	Siemens Healthineers	ACUSON S2000	18L6 high density	9–16	Uni/bilateral	Yes	Halo	Yes	6 months

NA: not assessed.

### Evaluation of bias

**Fig. 2:**
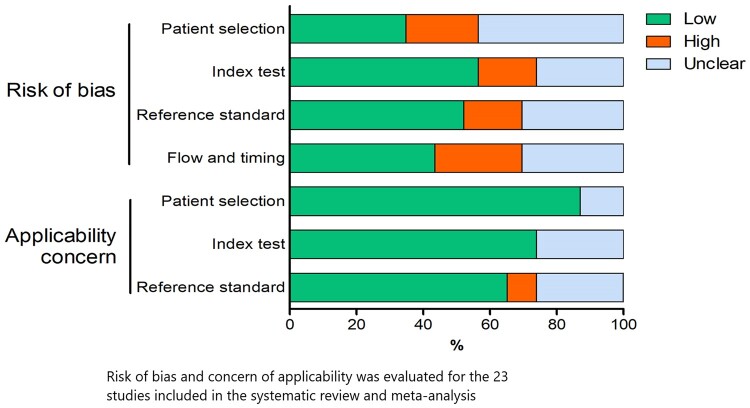
Overall Summary of QUADAS-2 items

Patient selection and flow of timing were the primary sources of bias (Fig. 2). Studies using TAB as the reference standard might have contributed to the selection bias, because there would be a strong initial clinical suspicion to request this invasive test. Studies using ACR 1990 clinical criteria as the diagnosis standard were at high risk of bias, because the index test could have altered the initial clinical decision. The flow of timing had a considerable amount of risk of bias, because the index test was performed at various time periods from the initial clinical suspicion of GCA. Additional data and details on the risk of bias assessment are summarized in Fig. 2 and Supplementary Fig. S2, available at *Rheumatology Advances in Practice* online. For the QUADAS-2 scale for diagnostic accuracy studies, the quality is reported in Supplementary Table S3, available at *Rheumatology Advances in Practice* online.

### Meta-analysis

**Fig. 3:**
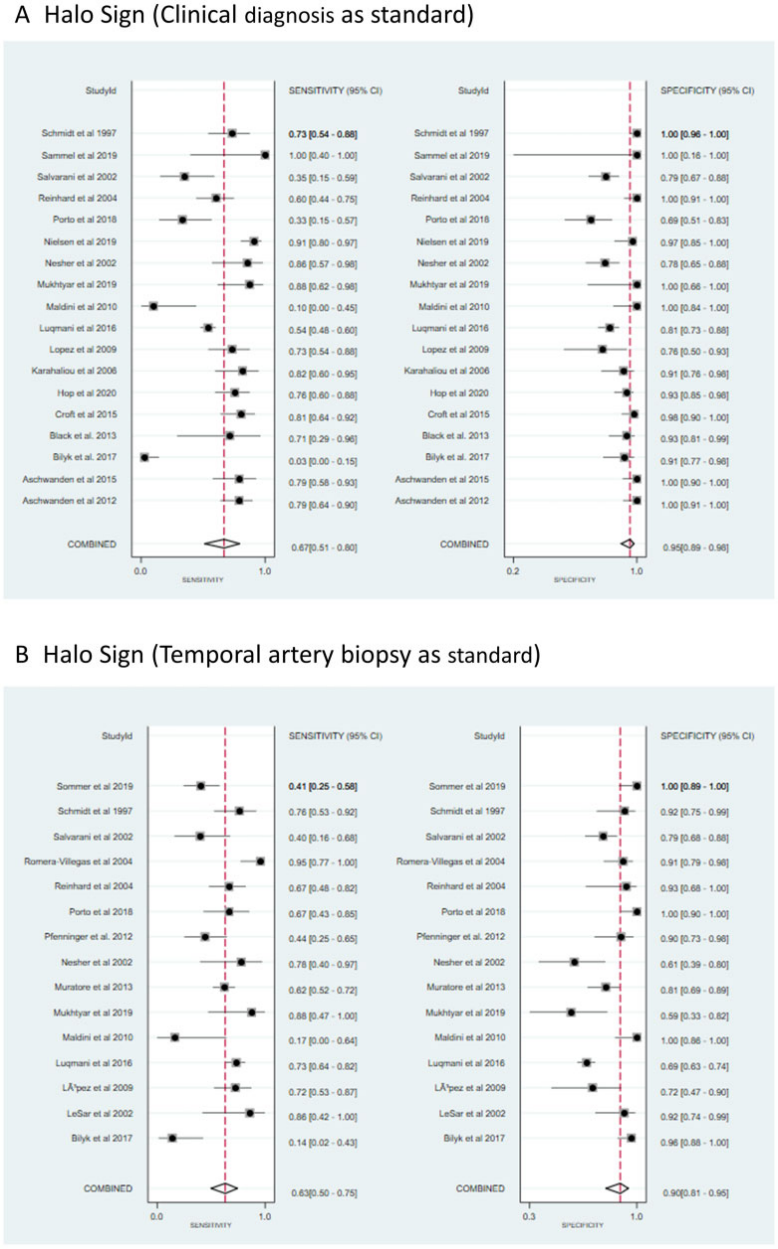
Forest plot of the sensitivity and specificity of the temporal artery US-derived halo sign for GCA (**A**) Studies with the clinical diagnosis as the reference standard for GCA. Temporal artery biopsy was performed in part of these studies. (**B**) Studies with the temporal artery biopsy as the reference standard for GCA.

Results of the pooled estimates for US signs of GCA in comparison to the clinical diagnosis or TAB as reference standard are summarized in Table 3. All 23 studies (*n* = 2711 patients) investigated the value of the halo sign in comparison to the clinical diagnosis ± TAB, yielding a pooled sensitivity of 67% (95% CI: 51, 80) and a specificity of 95% (95% CI: 89%, 98%). This gave a positive and negative likelihood ratio for the diagnosis of GCA of 14.2 (95% CI: 5.7, 35.5) and 0.35 (95% CI: 0.22, 0.54), respectively (Fig. 3A). When analysed, the halo sign with TAB as standard yielded a pooled sensitivity of 63% (95% CI: 50, 75) and a specificity of 90% (95% CI: 81, 95). The halo sign against TAB as standard revealed a positive likelihood ratio of 6.06 (95% CI: 3.34, 11.0) and a negative likelihood ratio of 0.41 (95% CI: 0.30, 0.56) (Fig. 3B). The analysis of the combined US signs (halo sign, stenosis or occlusion) in comparison to clinical diagnosis or TAB (four studies, *n* = 270) resulted in a sensitivity of 52% (95% CI: 18, 84) and specificity of 81% (95% CI: 64, 91) (Supplementary Fig. S3A, available at *Rheumatology Advances in Practice* online). The combination of halo sign and stenosis (four studies, *n* = 230) resulted in a sensitivity of 43% (95% CI: 12, 80) and specificity of 85% (95% CI: 66, 94) (Supplementary Fig. S3B, available at *Rheumatology Advances in Practice* online). Authors of two studies (*n* = 140, both with low risk of bias), from the same research group, investigated the compression sign [24, 40] and described sensitivities between 77 and 79% and a specificity of 100% of the compression sign when compared with the clinical diagnosis of cranial GCA. When comparing the studies done before 2010 (seven studies) and after 2010 (11 studies), later studies showed higher sensitivity of 71% (earlier studies, 63%) and similar specificity 96% (earlier studies, 95%) (Supplementary Table S4, available at *Rheumatology Advances in Practice* online).

**Table 3:** Meta-analysis of diagnostic accuracy of US signs for a diagnosis of GCA

Index test	Reference standard	Number of patients	Number of studies	Positive likelihood ratio (95% CI)	Negative likelihood ratio (95% CI)	Sensitivity, % (95% CI)	Specificity, % (95% CI)	DOR (95% CI)
Halo sign	Clinical diagnosis ±TAB	1502	18	14.21 (5.7, 35.5)	0.35 (0.22, 0.54)	67 (51-80)	95 (89, 98)	40.9 (12.1, 137.5)
Halo sign	TAB	1209	15	6.06 (3.34, 11.0)	0.41 (0.30, 0.56)	63 (50, 75)	90 (81, 95)	14.7 (7.3, 29.6)
Halo sign ± stenosis ± occlusion	Clinical diagnosis/TAB	270	4	2.70 (0.71, 10.26)	0.60 (0.24, 1.51)	52 (18, 84)	81 (64, 91)	4.5 (0.48, 42.6)
Halo sign ± stenosis	Clinical diagnosis/TAB	230	4	2.92 (0.90, 9.46)	0.67 (0.73, 3.04)	43 (12, 80)	85 (66, 94)	4.4 (0.71, 26.6)

A temporal artery biopsy was also performed in some studies with the clinical diagnosis as the reference standard for GCA. Clinical diagnosis is the final diagnosis made according to the ACR criteria or physician diagnosis. DOR: diagnostic odds ratio; TAB: temporal artery biopsy.

Forest plots and HSROC curves indicated that clinical diagnosis or TAB as a standard had limited heterogeneity, whereas halo sign with stenosis and occlusion or halo with stenosis showed high between-study heterogeneity (Supplementary Fig. S1, available at *Rheumatology Advances in Practice* online).

## Discussion

This systematic review and meta-analysis evaluated the role of the halo sign in the assessment of GCA. When compared with previous meta-analysis, the diagnostic performance of the halo sign for the diagnosis of cranial GCA was of similar sensitivity (67% *vs* 68–77%) [19, 22, 23, 56], but higher specificity (95% *vs* 81–96%) [19, 22, 23, 56]. When combining the halo sign with occlusion or stenosis, the present study showed lower sensitivity (52% *vs* 78%) [56] and higher specificity (81% *vs* 79%) [56]. This discrepancy could be attributable to the inclusion of high-quality studies and exclusion of overlapping studies, and might also be related to better equipment, with 5–15 MHz probes used in the earlier studies. Another reason could be that occlusion and stenosis are not assessed routinely, as mentioned in OMERACT, and more work is certainly needed to standardize the definition of these findings. A recent study showed that when combining the GCA pre-test probability score with the halo sign, the sensitivity increases to between 94 and 100% [57].

The present study also showed a comparable diagnostic accuracy of the halo sign compared with TAB. US might be a more thorough GCA assessment than TAB, because it allows for detailed analysis of the temporal arteries along their entire length, minimizing the effect of skip lesions [58]. TAB is also an invasive procedure, which can have procedural complications, and is not readily available for re-assessment of the artery if relapse occurs. In line with these findings, a review by Schmidt *et al.* [59] reported that biopsy has a relatively low yield compared with US in GCA diagnosis. The statistical findings of the present study indicate that the halo sign is a useful tool that could be incorporated in everyday clinical practice, because US is cost-effective and provides more accurate and specific results for the assessment of GCA. The findings of the TABUL study provided significant results for the specificity and sensitivity of the halo sign in GCA assessment, with values of 69% and 82%, respectively [15]. It asserts that the use of US in GCA assessment is highly dependent on the halo sign, because it determines the presence of an area of inflammation in the arteries. A recent publication of the novel halo score, graded with the halo thickness, confirms that the halo sign and halo count are significantly correlated with inflammatory markers, ocular ischaemia and intimal hyperplasia on TAB [3].

Limitations of this systematic review and meta-analysis are the inclusion of both prospective and retrospective observational studies. The retrospective studies might have contributed to bias in analysis of the final data. It has not been possible to evaluate the specific issues related to US operator and image interpretation variability [60]. The reviews did not present inter-rater/intra-rater reliability data. Different sonographic skill levels of the rheumatologists or sonographers might have had an impact on the final results. When the colour intensity is more robust, such as in smaller vessels, it is easier to distinguish the dark, hypoechoic halo sign [56]. Other malignant conditions, ANCA vasculitis, infections or poor US technique, can give rise to a false-positive halo [52]. A further issue was the methodologies used between the studies. Studies concluding that US is superior to TAB in diagnosis of GCA vary in their design [35, 46]. We included studies if they had US performed >2 weeks from the initial clinical suspicion of GCA, although they would have been exposed to a high dose of CSs, which might reduce the halo thickness and accuracy of US. When the ACR classification criteria for GCA were applied as the reference standard [22, 61], the meta-analyses reported a lower sensitivity and a higher specificity of the halo sign for GCA diagnosis. However, these criteria were designed for classification and research purposes and are inadequate for diagnosis of GCA in clinical practice [21]. Therefore, ACR criteria as the reference standard could be a limiting factor in the present study.

### Conclusion

This meta-analysis shows that the US halo sign has a significant role in the assessment and diagnosis of GCA. US is a sensitive and specific approach for GCA assessment, which seems to be improving with better equipment and user familiarity with US techniques. However, the studies analysed showed heterogeneity in their design and outcomes. Therefore, it is recommended that future researchers conduct multicentre prospective studies for analysing the effectiveness of the halo sign in the assessment of GCA, with a standardized study protocol.

Study concept and design: A.S., F.C., K.S.M.v.d.G., S.I. and B.D.; data collection: A.S. and F.C.; statistical analysis and data interpretation: A.S., F.C., J.J. and K.S.M.v.d.G.; all authors reviewed the manuscript content and gave the final approval of the version.

## Supplementary Material

rkab059_Supplementary_Data

## Data Availability

All data relevant to the study are included in the article. All authors agree to make materials, data and associated protocols promptly available to readers if requested.
