# Supplementary material for: Role of the halo sign in the assessment of giant cell arteritis: a systematic review and meta-analysis
Source: Rheumatol Adv Pract. 2021 Aug 19;5(3):rkab059. doi: 10.1093/rap/rkab059 (PMC8421813; doi:10.1093/rap/rkab059)
Supplement: rkab059_Supplementary_Data [file rkab059_supplementary_data.zip › 21-055 Supplementary Table S3.docx]

**Supplementary Table S3.** **QUADAS-2 tool guidance for authors**

| **DOMAIN** | **Yes** | **No** |  |  |  | **Unclear** |
| --- | --- | --- | --- | --- | --- | --- |
|  |  | | | | | |
| **PARTICIPANT** | **Describe methods of participant selection: participants with symptoms of suspected GCA in** | | | | | |
| **SELECTION** | **Primary or secondary care with no prior testing for GCA except for blood testing.** | | | | | |
|  |  |  | | | |  |
| **Was a consecutive or ran-** | Consecutive sampling or ran- | Non-random sampling or sam- | | | | Unclear whether consecutive or |
| **dom sample of participants** | dom sampling of participants | pling based on volunteering or | | | | random sampling used |
| **enrolled?** | according to inclusion criteria | referral |  |  |  |  |
|  |  |  | | | |  |
| **Was a case-control design** | One gate design: GCA suspects | Two gate design: diseased and | | | | Unclear study design: These |
| **avoided?** | included based on a single set | non-diseased patients included | | | | studies will be excluded. |
|  | of inclusion criteria. All studies | based | on | different | criteria, |  |
|  | will be in this category given our | These studies will be excluded | | | |  |
|  | inclusion criteria |  |  |  |  |  |
|  |  |  | | | |  |
| **Did the study avoid inappro-** | Exclusions are detailed and felt | Inappropriate exclusions, such | | | | Reporting insufficient to assess |
| **priate exclusions?** | to be appropriate (e.g. pre-exist- | as “difficult-to-diagnose pa- | | | | this item |
|  | ing diagnosis of GCA) | tients”, are reported | | |  |  |
|  |  |  | | | |  |
| **Risk of bias: did the selec-** | Yes, for both signalling ques | No for one or two sig- | | | | Unclear for one or two sig- |
| **tion of participants avoid in-** | tions: participants recruited in | nalling | questions: participants | | | nalling questions: reporting in- |
| **troduction of bias?** | a consecutive manner with ap- | recruited in a non-consecu- | | | | sufficient to assess this item |
|  | propriate exclusions only | tive manner with inappropriate | | | |  |
|  |  | and/or unspecified exclusions | | | |  |
|  |  |  | |  |  |  |
| **Concerns regarding applica-** | Inclusion of adult participants | Population | | included | does not | Unclear inclusion criteria |
| **bility: did participants in-** | with suspected GCA | reflect that of adult participants | | | |  |
| **cluded in the study appropri-** |  | with suspected GCA | | |  |  |
| **ately match the review ques-** |  |  |  |  |  |  |
| **tion?** |  |  |  |  |  |  |
|  |  | | | | | |
| **INDEX TESTS** | **Temporal artery ultrasound performed by a trained ultra-sonographer, and temporal artery** | | | | | |
|  | **biopsy** |  |  |  |  |  |

|  |
| --- |
|  |

**Supplementary Table S3.** **QUADAS 2 guidance for authors** (*Continued)*

**Were the index test results interpreted without knowledge of the results of the reference standard?**

Index tests performed in a double-blinded setting or prior to knowledge of results of reference standard

Index tests performed post-ref-erence standard outcome in non-double blinded setting

Unclear reporting of index test blinding

| **If a threshold was used, was it** | | | Temporal | artery ’halo sign’ | Threshold not pre-specified | | Unclear whether threshold is |
| --- | --- | --- | --- | --- | --- | --- | --- |
| **pre-specified?** | |  | thickness reported to be pre- | |  |  | pre-specified or not |
|  |  |  | specified or available in a study | |  |  |  |
|  |  |  | protocol |  |  |  |  |
|  | | |  | |  | |  |
| **Was the test appropriately ex-** | | | Clear definition of objective cri- | | ’Halo sign’ not clearly defined | | Insufficient evidence to support |
| **ecuted and a clear definition** | | | teria to define positive ’halo | | and | subjectively determined | or refute |
| **of positivity provided (tem-** | | | sign’. Imaging scans’ quality was | | by ultra-sonographer. Imaging | |  |
| **poral artery ultrasound)?** | | | assessed. Appropriate operator | | scans’ | quality assessment not |  |
|  |  |  | training and equipment used | | used, i.e. no selection made | |  |
|  |  |  |  |  | based on image quality. Evi- | |  |
|  |  |  |  |  | dence | of suboptimal operator |  |
|  |  |  |  |  | training and equipment | |  |
|  |  | |  | |  | |  |
| **Was the** | **test appropriately** | | Biopsy length, time frame for | | Quality assessment not used | | Reporting insufficient to assess |
| **executed?** | **(temporal** | **artery** | collection | and sample quality |  |  | this item (e.g. biopsy length or |
| **biopsy)** |  |  | was assessed and the relative cri- | |  |  | time frame not mentioned) |
|  |  |  | teria are clearly reported | |  |  |  |
|  | |  |  | |  | |  |
| **Concerns regarding** | | **applica-** | The test procedure is one that | | Ultrasonography on alternative | | Unclear tests or unclear study |
| **bility: are there concerns that** | | | is used in current practice and | | blood vessels rather than tem- | | personnel profile, background |
| **the index tests, their conduct,** | | | the training of staff in the study | | poral artery. Tests are not vali- | | and training |
| **or interpretation differ from** | | | reflects the training of staff that | | dated or study personnel is in- | |  |
| **the review questions?** | |  | would be required in current | | sufficiently trained | |  |
|  |  |  | practice |  |  |  |  |
|  | | |  | | | | |
| **REFERENCE STANDARD** | | | **Describe the reference standard and how it was conducted and interpreted.** | | | | |
|  |  |  |  |  |  |  |  |

**Is the reference standard likely to correctly classify the target condition?**

Use of ACR 1990 Classification for diagnosis of GCA consistent and appropriate

| Inconsistent or heterogeneous | Reporting insufficient to assess |
| --- | --- |
| use of component tests within | this item |
| the ACR 1990 Classification for |  |
| diagnosis of GCA |  |

**Were the reference standard results interpreted without knowledge of the results of the index test?**

Reference standard performed ’blinded’ or ’independently and without knowledge of ’ index test, results are sufficient and full details of the blinding procedure are not required; or clear temporal pattern to the order of testing that precludes the need for formal blinding

Reference standard was performed and assessed with knowledge of the results of index tests

Unclear whether results were interpreted independently

|  |
| --- |

**Supplementary Table S3.** **QUADAS 2 guidance for authors** (*Continued)*

**Risk of bias: could the refer-ence standard, its conduct, or its interpretation have intro-duced bias?**

Criteria for signaling questions above adequately met. Use of the ACR 1990 Classification performed without knowledge of index test results

Criteria for signaling ques-tions above not adequately met. Inconsistent use of ACR 1990 Classification, or tests per-formed with knowledge of index test results

Unclear reports of conduct or interpretation of the reference standard

**Concerns regarding applica-bility: are there no concerns that the target condition as defined by the reference stan-dard does not match the re-view question?**

Use of ACR 1990 Classification for diagnosis of GCA consistent and appropriate, maximising diagnostic accuracy of tar-get condition

Use of ACR 1990 Classifica-tion for diagnosis of GCA in-consistent leading to question-able diagnostic accuracy for tar-get condition

Unclear or heterogeneous diagnoses

| **FLOW AND TIMING** | **Describe any participants who did not receive the index test(s) and/or reference standard or** | | | | | |
| --- | --- | --- | --- | --- | --- | --- |
|  | **who were excluded from the 2 x 2 table (refer to flow diagram): describe the time interval and** | | | | | |
|  | **any interventions between index test(s) and reference standard.** | | | | |  |
|  |  |  |  | | |  |
| **Was there an appropriate in-** | Time interval between | index | More than one week between | | | Unclear delay between execu- |
| **terval between index test(s)** | and reference test was less than | | index and reference test execu- | | | tion of index tests and reference |
| **and reference standard?** | one week |  | tion, or post initiation of treat- | | | standard |
|  |  |  | ment for GCA | |  |  |
|  |  | |  |  | |  |
| **Did all participants receive a** | All participants receiving the in- | | Not | all participants verified | | Unclear whether all participants |
| **reference standard?** | dex test were verified with the | | with reference standard | | | receiving the index test were |
|  | reference standard |  |  |  |  | verified with the reference stan- |
|  |  |  |  |  |  | dard |
|  |  | |  | | |  |
| **Did all participants receive** | ACR 1990 Classification is the | | ACR 1990 Classification ws not | | | Unclear whether ACR criteria |
| **the same reference standard?** | only reference standard allowed | | used. No studies will be in | | | were used. No studies will be |
|  | in this review. All studies will be | | this category given our inclu- | | | in this category given our inclu- |
|  | in this category given our inclu- | | sion criteria | |  | sion criteria |
|  | sion criteria |  |  |  |  |  |
|  |  | |  | | |  |
| **Were all participants included** | All participants enrolled in the | | Less than the whole sample en- | | | Reporting insufficient to assess |
| **in the analysis?** | study was included in the anal- | | rolled and included in the study | | | this item (e.g. some partici- |
|  | ysis |  | were included in the final anal- | | | pants’ exclusion was mentioned |
|  |  |  | ysis |  |  | but no specific details were re- |
|  |  |  |  |  |  | ported) |
|  |  | |  | | |  |
| **Risk of bias: did the partici-** | Criteria for signaling questions | | Criteria for signalling questions | | | Reporting insufficient to assess |
| **pantflow avoid the introduc-** | above adequately met. Clear | | above not adequately met. Un- | | | this item |
| **tion of bias?** | timeline of participant | flow | clear | timeline | of participant |  |
|  | with an interval of ≥ 1 week be- | | flow | with an | interval of > |  |
|  | tween index test and reference | | 1 month between index test | | |  |
|  | standard. No prior steroid use | | and reference standard, or prior | | |  |
|  |  |  | steroid use | |  |  |

**Supplementary Table 3.** **QUADAS 2 guidance for authors** (*Continued)*

**DIRECT COMPARATIVE** **Describe the sequence of testing adopted in studies.**

**STUDIES**

| **Were ultrasound results inter-** | Index tests performed in a | Examiners or pathologists were |
| --- | --- | --- |
| **preted without knowledge of** | blinded setting | aware of the other index test re- |
| **the results of both biopsy and** |  | sults |
| **vice versa?** |  |  |

Unclear reporting of ultrasound blinding to biopsy results and the reference standard

**Were biopsy results inter-preted without knowledge of the results of both ultrasound and the reference standard?**

***ACR****: American College of Rheumatology;* ***GCA****: giant cell arteritis*

Biopsy specimen assessment performed in a blinded setting to the results of both ultrasound and reference standard

Examiner assessing biopsy spec-imen aware of diagnosis or post-reference standard outcome in non-blinded setting

Unclear reporting of biopsy blinding to ultrasound and ref-erence standard
