# Supplementary material for: Role of the halo sign in the assessment of giant cell arteritis: a systematic review and meta-analysis
Source: Rheumatol Adv Pract. 2021 Aug 19;5(3):rkab059. doi: 10.1093/rap/rkab059 (PMC8421813; doi:10.1093/rap/rkab059)
Supplement: rkab059_Supplementary_Data [file rkab059_supplementary_data.zip › 21-055 Supplementary Material.docx]

**Supplementary figure S1:** **Summary receiver operating characteristic (sROC) curves of the temporal artery ultrasonography derived halo sign compared to the final diagnosis of GCA.**

Each ellipse corresponds to a study estimate of sensitivity and specificity; the area of each ellipse is proportional to study size. Dotted lines indicate unweighted analysis; black lines indicate weighted analysis. Shaded rectangles mark the 95% CI of the pooled sensitivity and pooled specificity by random-effects calculation. Exact estimates indicated by a black dot.


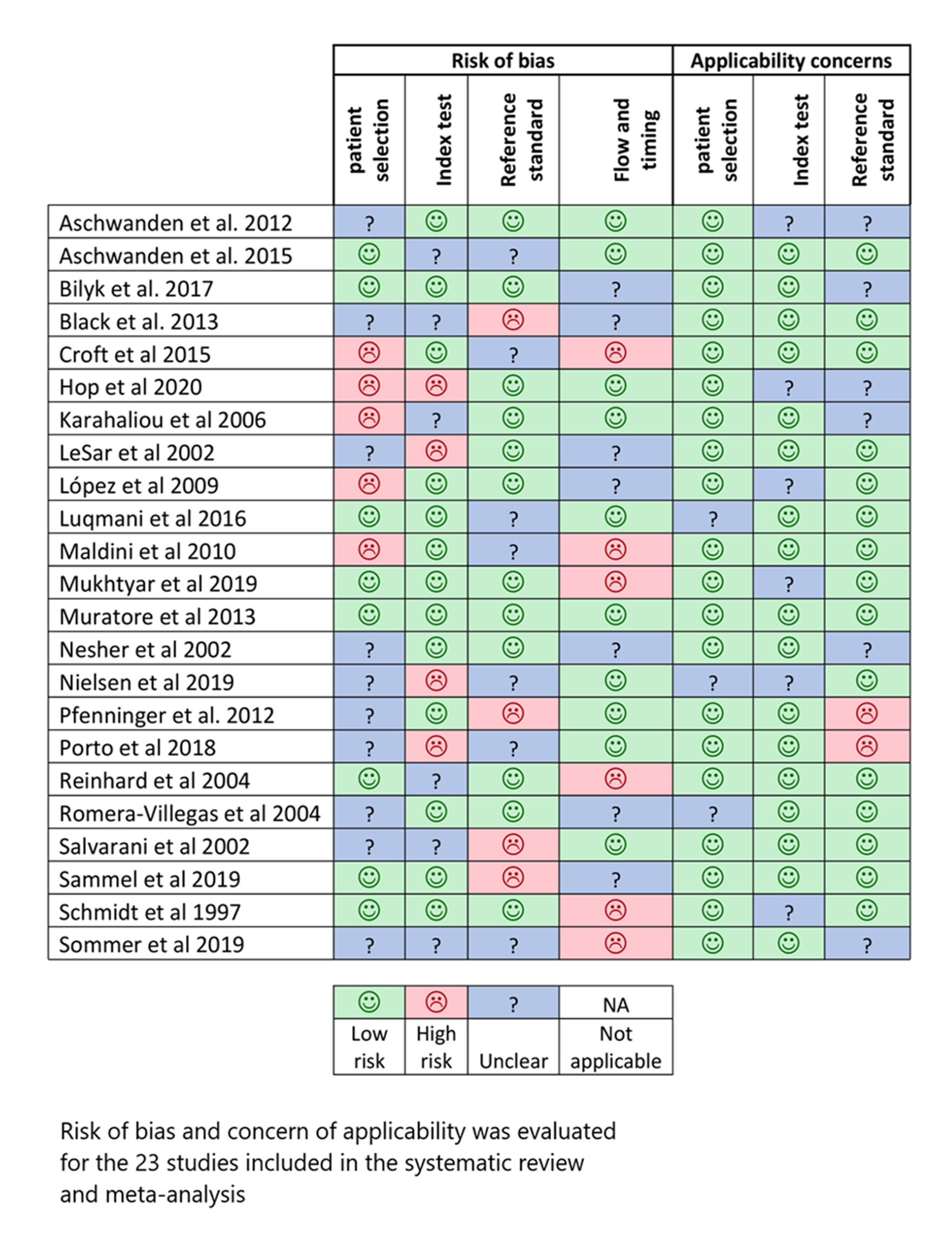


**Supplementary Figure S2. Detailed summary of QUADAS-2 Items**

**Supplementary Figure S3. Analysis of Halo sign**

**Supplementary Table S1. Search strategy- PICO**

|  | |
| --- | --- |
| population | Adult human subjects (age 50 years and above clinically classified as suspected GCA.  These participants must have had temporal artery and/or axillary artery US to look for the halo sign. Participants with suspected GCA can include those with a prior diagnosis of polymyalgia rheumatica |
| intervention | ultrasound, (**Halo Sign, Compression sign, Stenosis, Occlusion)**  **Temporal artery US**: definitions have been developed through the work of the Outcome Measures in Rheumatology (OMERACT) Large Vessel Vasculitis (LVV) US Working Group |
| comparator | Clinical diagnosis temporal artery biopsy, MRI/angiography, CT/angiography and PET / CT |
| outcome | Main Outcome (s): Accuracy of the ‘halo sign’ in diagnosing GCA  i.e. sensitivity, specificity, diagnostic odds ratio, positive likelihood ratio, negative likelihood ratio, accuracy |

**Supplementary Table S2. SEARCH STRATEGY- Key words**

**Keywords**

| giant cell arteritis | "giant cell arteritis"[MeSH Terms] OR ("giant"[All Fields] AND "cell"[All Fields] AND "arteritis"[All Fields]) OR "giant cell arteritis"[All Fields] |
| --- | --- |
| temporal arteritis | "giant cell arteritis"[MeSH Terms] OR ("giant"[All Fields] AND "cell"[All Fields] AND "arteritis"[All Fields]) OR "giant cell arteritis"[All Fields] OR ("temporal"[All Fields] AND "arteritis"[All Fields]) OR "temporal arteritis"[All Fields] |
| cranial arteritis | "giant cell arteritis"[MeSH Terms] OR ("giant"[All Fields] AND "cell"[All Fields] AND "arteritis"[All Fields]) OR "giant cell arteritis"[All Fields] OR ("cranial"[All Fields] AND "arteritis"[All Fields]) OR "cranial arteritis"[All Fields] |
| ultrasound | "diagnostic imaging"[Subheading] OR ("diagnostic"[All Fields] AND "imaging"[All Fields]) OR "diagnostic imaging"[All Fields] OR "ultrasound"[All Fields] OR "ultrasonography"[MeSH Terms] OR "ultrasonography"[All Fields] OR "ultrasound"[All Fields] OR "ultrasonics"[MeSH Terms] OR "ultrasonics"[All Fields] |
| ultrasonography | "diagnostic imaging"[Subheading] OR ("diagnostic"[All Fields] AND "imaging"[All Fields]) OR "diagnostic imaging"[All Fields] OR "ultrasonography"[All Fields] OR "ultrasonography"[MeSH Terms] |

**Search string**

(("giant cell arteritis"[MeSH Terms] OR ("giant"[All Fields] AND "cell"[All Fields] AND "arteritis"[All Fields]) OR "giant cell arteritis"[All Fields]) OR GCA[All Fields] OR ("giant cell arteritis"[MeSH Terms] OR ("giant"[All Fields] AND "cell"[All Fields] AND "arteritis"[All Fields]) OR "giant cell arteritis"[All Fields] OR ("temporal"[All Fields] AND "arteritis"[All Fields]) OR "temporal arteritis"[All Fields]) OR ("giant cell arteritis"[MeSH Terms] OR ("giant"[All Fields] AND "cell"[All Fields] AND "arteritis"[All Fields]) OR "giant cell arteritis"[All Fields] OR ("cranial"[All Fields] AND "arteritis"[All Fields]) OR "cranial arteritis"[All Fields])) AND (("diagnostic imaging"[Subheading] OR ("diagnostic"[All Fields] AND "imaging"[All Fields]) OR "diagnostic imaging"[All Fields] OR "ultrasound"[All Fields] OR "ultrasonography"[MeSH Terms] OR "ultrasonography"[All Fields] OR "ultrasound"[All Fields] OR "ultrasonics"[MeSH Terms] OR "ultrasonics"[All Fields]) OR ("diagnostic imaging"[Subheading] OR ("diagnostic"[All Fields] AND "imaging"[All Fields]) OR "diagnostic imaging"[All Fields] OR "ultrasonography"[All Fields] OR "ultrasonography"[MeSH Terms]) OR duplex[All Fields] OR halo[All Fields])

("Halo sign"[Title/Abstract] OR "US"[Title/Abstract] OR "Ultrasound"[Title/Abstract] OR "Sonograpgh"[Title/Abstract] OR ("Giant cell arteritis"[Title/Abstract] OR "temporal arteritis"[Title/Abstract] OR "LVV"[Title/Abstract] OR "Hrton disease"[Title/Abstract] OR "vasculitis"[Title/Abstract] OR "GCA"[Title/Abstract] OR "Occlusion"[Title/Abstract] OR "stenosis"[Title/Abstract] OR "axillary artery"[Title/Abstract])

**Cochrane**

1. Giant Cell Arteritis/

2. (giant adj2 cell adj2 arteritis).tw.

3. ((temporal or cranial) adj2 arteritis).tw.

4. GCA.tw.

5. or/1-4

6. exp Ultrasonography/

7. Laser-Doppler Flowmetry/

8. (ultrasou$ or sonogra$ or biomicroscop$).tw.

9. (halo or halos).tw.

10. (doppler or duplex).tw.

11. (TAU or UBM).tw.

12. Temporal Arteries/dg [Diagnostic Imaging]

13. or/6-12

14. 5 and 13

**MEDLINE /Embase Ovid search strategy**

1. giant cell arteritis/

2. temporal arteritis/

3. (giant adj2 cell adj2 arteritis).tw.

4. ((temporal or cranial) adj2 arteritis).tw.

5. GCA.tw.

6. or/1-5

7. exp echography/

8. exp echograph/

9. (ultrasou$ or sonogra$ or biomicroscop$).tw.

10. (halo or halos).tw.

11. (doppler or duplex).tw.

12. (TAU or UBM).tw.

13. or/7-12

14. 6 and 13

**PubmedCENTRAL search strategy**

#1 MeSH descriptor: [Giant Cell Arteritis] explode all trees

#2 giant near/2 cell near/2 arteritis

#3 (temporal or cranial) near/2 arteritis

#4 GCA

#5 #1 or #2 or #3 or #4

#6 MeSH descriptor: [Ultrasonography] explode all trees

#7 MeSH descriptor: [Laser-Doppler Flowmetry] this term only

#8 (ultrasou* or sonogra* or biomicroscop*)

#9 (halo or halos)

#10 (doppler or duplex)

#11 TAU or UBM

#12 MeSH descriptor: [Temporal Arteries] this term only and with qualifier(s): [diagnostic imaging - DG]

#13 #6 or #7 or #8 or #9 or #10 or #11 or #12

#14 #5 and #13

**Please note: Supplementary Table S3 is available in a separate document.**

**Supplementary Table S4. Results of sensitivity/Specificity analysis of studies, Early (before 2010) vs later (after 2010)**

| **Studies** | **Number of studies** | **Sensitivity% (95% CI)** | **Specificity % (95% CI)** | **LR+ (95% CI)** | **LR- (95% CI)** | **DOR (95% CI)** |
| --- | --- | --- | --- | --- | --- | --- |
| **Early (before 2010)** | 7 | 63 (42-80) | 96 (78-99) | 15.4 (2.59-91.78) | 0.39 (0.23-0.65) | 39.60 (5.95-263.67) |
| **Later (After 2010)** | 11 | 71 (49-86) | 95 (88-98) | 15.5 (5.06-47.4) | 0.31 (0.16-0.61) | 50.2 (9.5-264.0) |

CI, confidence interval; DOR, Diagnostic odds ratio; LR, likelihood ratio.
